# Moving online: reflections from conducting system dynamics workshops in virtual settings

**DOI:** 10.1002/sdr.1667

**Published:** 2020-12-01

**Authors:** Nici Zimmermann, Irene Pluchinotta, Giuseppe Salvia, Marianne Touchie, Helen Stopps, Ian Hamilton, Ted Kesik, Kaveh Dianati, Ting Chen

**Affiliations:** ^1^ UCL Institute for Environmental Design and Engineering University College London Gower Street London WC1E 6BT UK; ^2^ Department of Mechanical and Industrial Engineering University of Toronto 5 King's College Road Toronto ON M5S 3G8 Canada; ^3^ Department of Civil and Mineral Engineering University of Toronto 35 St. George Street Toronto ON M5S 1A4 Canada; ^4^ UCL Energy Institute University College London Gower Street London WC1E 6BT UK; ^5^ John H. Daniels Faculty of Architecture, Landscape, and Design University of Toronto 1 Spadina Crescent Toronto Toronto ON M5S 2J5 Canada

## Introduction

Due to the COVID‐19 pandemic, it has become increasingly important and necessary to conduct research and teaching activities online. While many universities quickly started the transition to online teaching, most in‐person research activities were typically postponed. However, in order to not significantly delay research, researchers are increasingly seeking to conduct activities, such as workshops, in a virtual setting. Following this initial period of disruption, it is expected that many online or hybrid activities will continue to be used and, in some cases, may replace in‐person interactions due to cost, time, convenience, and environmental concerns after the COVID‐19 crisis has passed. Researchers and/or stakeholders are often geographically dispersed, which independently motivates the use of online or hybrid workshop activities.

Different types of online activities and tools related to system dynamics have existed for some time. They include interactive simulation environments such as Climate Interactive's widely used C‐ROADS and En‐ROADS models (e.g. Rooney‐Varga *et al*., 2020) as well as purpose‐built solutions for single workshops (e.g. Eker *et al*., 2018). There is online system dynamics training (https://www.systemdynamics.org/online-course-catalog) and programmes (Pavlov *et al*., 2014), and researchers have reported about online peer mentoring groups (Richardson *et al*., 2015; Suprun *et al*., 2020). The 2020 International Conference of the System Dynamics Society ran online and included a workshop by Michael Bean on delivering online simulations, particularly in teaching contexts. In addition, fuelled by the effects of COVID‐19, the idea of online system dynamics workshops is gaining prominence. Virtual delivery of workshops has been reported sparsely in the areas of problem‐structuring methods and group‐support systems (Yearworth and White, 2017, 2019), and there is a single recent contribution in the area of system dynamics (Wilkerson *et al*., 2020). It is time to further discuss the practicalities of moving workshops online and make some recommendations for effective online workshop delivery.

Here, we report on three cases of conducting system dynamics workshops online: first, confirmatory/disconfirmatory workshops that included online participants because of an internationally diverse project team; second, an En‐ROADS Climate Workshop that was part of an online system dynamics lecture; and third, group model building (GMB) and project prioritisation workshops moved online in response to social‐distancing restrictions during the COVID‐19 lockdown. We describe these three cases and insights derived from them for online system dynamics workshop delivery and then discuss implications and future research avenues.

## Cases

This section presents the three rather different case settings. We present them chronologically from a first international online workshop in 2018 to recent activities in 2020.^i^

### Confirmatory/disconfirmatory workshops on tower block refurbishment

In October 2018 and June 2019, we conducted two confirmatory/disconfirmatory workshops which included online participation and had seven to eight participants, excluding the facilitator(s). The workshops were part of an international research project that developed recommendations for refurbishments of residential tower blocks to improve their energy efficiency and involved team members from Canada and the United Kingdom (see Stopps *et al*., 2020). The confirmatory/disconfirmatory workshop setting serves validation purposes and is inspired by the disconfirmatory interviews (Andersen *et al*., 2012) and the Model Review script (Scriptapedia Wikibooks contributors, n.d.). It presents an existing causal loop diagram (CLD) to participants and asks them for structure confirmation or improvement and/or adaptation to a slightly different (e.g. geographical) context.

The first workshop served to review U.K.‐based CLDs and to investigate similarities and differences to the Canadian context. It lasted around five hours and was facilitated by a team member via Skype for Business software from London. Three further project team members participated online, and two team members and two Canadian practitioners participated jointly from a meeting room in Toronto. We slowly unfolded and walked through five existing CLDs that represented the U.K. context of tower refurbishment, and the group discussed similarities and differences to the Canadian context (see *Table* 1). This first workshop gave Canadian participants a sound understanding of the U.K. context and its complexity and was thus a great preparation for the second workshop.

**Table 1:** Participant agenda of the first tower‐refurbishment workshop

First tower‐refurbishment workshop (5 hours)	
15 minutes	Introduction of participants and purpose
60 minutes	Guidance CLD Presentation of the causal loop diagram Clarification and improvement of the diagram
30 minutes	Product compliance CLD Presentation of the causal loop diagram Clarification and improvement of the diagram
15 minutes	*Break*
30 minutes	Oversight CLD Presentation of the causal loop diagram Clarification and improvement of the diagram
30 minutes	Supply chain fragmentation CLD Presentation of the causal loop diagram Clarification and improvement of the diagram
15 minutes	*Break*
45 minutes	Procurement CLD Presentation of the causal loop diagram Clarification and improvement of the diagram
45 minutes	Full CLD Presentation of the causal loop diagram Clarification and improvement of the diagram
15 minutes	Discussion of the use of the causal loop diagrams, next steps, and close

Eight months later, the U.K. project team hosted a second three‐hour workshop in London, joined online by the Toronto project team. It served the purpose of adding a resident perspective to four of the U.K.‐based CLDs, and it served stakeholders to make their perspective heard. We presented the CLDs to two subgroups of physically attending stakeholders from a Neighbourhood Forum, the community group alliance Just Space, and a resident‐managed social housing association. The Canadian colleagues attended online via laptops that were placed on each of the group tables at the London workshop location, and they were asked to be nondominant participants, just coming in occasionally with questions or comments based on their subject‐matter expertise. An overview of the agenda of this second workshop is given in *Table* 2, and details are described in the online supplement to this article.

**Table 2:** Participant agenda of the second tower‐refurbishment workshop

Second tower‐refurbishment workshop (3 hours)	
10 minutes	Introduction of participants
10 minutes	Presentation of the project and intermediate results
40 minutes	Small group work on first set of causal diagrams 10 minutes presentation of a diagram 30 minutes improvement of the diagram
30 minutes	Presentation of the results to the plenary and discussion
10 minutes	*Break*
40 minutes	Small group work on second set of causal diagrams 10 minutes presentation of a diagram 30 minutes improvement of the diagram
30 minutes	Presentation of the results to the plenary and discussion
10 minutes	Discussion and close

The lessons learned from running the first and second workshops online were as follows:

- *Workshop format*: Having a facilitator join online or do group work with a mix of in‐person and online participants is certainly unusual and demonstrates that a breadth of hybrid activities, that include some combination of participants together in a room and participants engaging in the workshop online, is possible.
- *Number of participants*: The small number of participants was very helpful for these hybrid settings and is recommended.
- *Workshop length*: The first 5‐hour workshop with Canadian colleagues and stakeholders was too long for an online setting. A shorter workshop would be preferred. This was tested in the reduced 3‐hour duration of the second workshop, which worked very well. To make more efficient use of the workshop time, we suggest having several facilitators to simultaneously run confirmatory/disconfirmatory activities on several CLDs in breakout groups and then share the findings (online) with the group.
- *Participants' focus*: Particularly in the beginning and at the end of the first workshop, the facilitator found it difficult to keep the participants focused on evaluating the causal structure. In the beginning, this might have been because of their lack of familiarity with the task and, in the end, it might have been because the discussion moved towards the wider context and participants wanted to bring in further points, or they were simply tired. The unusual setting of the facilitator being present remotely at the first workshop certainly aggravated the situation because it was difficult to focus the participants' attention to the screen in their room and away from a normal group discussion on a topic. It would be interesting to investigate whether this challenge can be addressed by having at least one facilitator for each geographic location.
- *Equal opportunities for participation*: Vennix (1996, pp. 165–166) discusses the problem of dominant talkers versus shy participants in GMB and the role of the facilitator in ensuring equal opportunities for participation. In a hybrid (in‐person and online) setting, we found that the generic risk of unequal participation can take on a new dimension as online participants who are not normally shy may find it difficult to know where to come into the conversation since they are not able to fully read physical cues from behind the screen and might therefore contribute less than they would have in a face‐to‐face setting. High‐quality technical set‐up where online participants can have a full view of the room while in‐person participants can also see all online participants could help mitigate this risk, but this may not always be possible. Vennix (1996, p. 166) suggests that structuring the discussion using techniques such the Nominal Group Technique (Vennix, 1996, p. 129) can help provide all participants a chance to contribute. Such structuring methods are perhaps even more important in a hybrid setting. During open, unstructured discussions, the facilitator needs to strike a balance between allowing lively in‐person debates to unfold organically versus interrupting to ensure that online participants are being sufficiently heard. In this regard, the *hand‐raising* feature of various online meeting platforms can also help, assuming a helper is available to continuously monitor the screen for raised hands.

Overall, the workshops allowed for cross‐country learning among participants, and they were very helpful for developing recommendations for refurbishments of residential tower blocks. While not perfect, the online setting allowed us to have a conversation which would otherwise not have been possible due to geographic distance.

### An online En‐ROADS climate workshop

Next, we report on a two‐hour En‐ROADS Climate Workshop that took place on 17 March 2020. The purpose of this workshop was to educate students about climate change and show them some of the breadth of system dynamics applications. It was part of a 3‐hour lecture of the Systems Thinking and System Dynamics module at University College London that had been moved online for COVID‐19‐related reasons. The lecture used Blackboard Collaborate Ultra software, which is a seminar teaching tool. It has similar functionality to a conference‐call software (e.g. audio and video participation, sharing slides or the entire screen, chat): it allowed students to raise their hands if they wanted to say something and the use of break‐out sessions during which subgroups of students could talk and chat among themselves.

The En‐ROADS Climate Workshop is a shorter version of the Climate Action Simulation Game and aims at creating awareness for climate change and the energy, economic growth, and land‐use policies needed to achieve climate goals (https://www.climateinteractive.org/tools/). This specific workshop used Climate Interactive's materials and followed many of their suggestions for in‐person workshop design (Jones *et al*., 2019). The workshop process is listed in *Table* 3.

**Table 3:** Process of the En‐ROADS Climate Workshop

En‐ROADS Climate Workshop (2 hours)		
20 minutes	Introductory presentation	The facilitator gave an introductory presentation on the climate crisis and the workshop task using Climate Interactive's and the facilitator's own slides (Climate Interactive, 2019).
5 minutes	Climate target agreement	The group agreed on a climate target to achieve, i.e. 2°C or 1.5°C warming. (This was woven into the introductory presentation.)
10 minutes	Task 1: Actions of close people	In breakout groups: What actions have you or your friends and neighbours done in the last 5 years to help mitigate climate change? (discussion in subgroups to come up with a list of actions)
5 minutes	Collection of responses	Responses were collected from the groups. (The facilitator wrote down notes on a sheet of paper in front of her to have the group responses visible when she started interacting with the En‐ROADS interface).
10 minutes	Simulation of responses	The facilitator repeated the shared responses and the group explored them on the En‐ROADS interface.
15 minutes	Task 2: Actions needed	In breakout groups: What else would it take to limit warming to less than 2°C or even 1.5°C? Please come up with a comprehensive list of suggestions. (discussion in subgroups to come up with a list of policies)
25 minutes	Collection of responses, simulation and discussion	Responses were collected from the groups, and the groups took turns exploring their suggestions on the interface in front of the larger group of participants. Results and further steps needed were discussed also concerning their feasibility.
25 minutes	Debriefing	Debriefing was conducted where the facilitator asked the group to either speak up or to use the chat when the facilitator asked them to reflect on specific questions.

The lessons learned from running the workshops online were as follows:

- *Workshop format*: The online setting worked very well for a workshop focused on agreeing on policies and simulating them.
- *Number of participants*: This teaching setting had a medium number of 10–12 participants. We think that this simulation type of workshop is flexible concerning participant numbers, as long as everybody has the chance to speak up in smaller break‐out groups and there is a way to share aggregated insights with the whole group.
- *Workshop length*: The workshop took place during the second and third hour of a three‐hour lecture. Limiting an online session to 2 hours would have been better.
- *Activity length*: It became clear that 10 minutes was almost too long for Task 1 where participants discussed what they, their friends, and their neighbours had done to mitigate climate change. A shorter session of 5 to 8 minutes would seem sufficient if subgroups are very small.
- *Technology available to each participant*: Not everybody participated with a microphone, so some students only contributed via the chat, making it feel a bit one‐sided towards the end. This may be due to the fact that it was only the second day of online teaching, and some students might not have had a microphone. It is also possible that students felt uncomfortable speaking knowing that the meeting was being recorded or generally still felt uncomfortable with the online teaching setting. It is thus important not to put students on the spot, to consider and remain flexible to participants' specific situation, to provide them with encouragement and multiple participation channels (e.g. microphone, chat), and not ask them to speak about themselves when in front of the whole group but to ask subgroups to share aggregated discussion information.
- *Software features*: In this specific software, the documents and chats from break‐out sessions got deleted once the session moved back to the full group. Thus, separate note taking and good documentation proved useful and necessary. Prior to the beginning of a workshop, it is thus useful to consider software features and constraints that limit information continuity or participation so their impacts may be mitigated.
- *Participants' autonomous use of the simulation model*: One subgroup found the En‐ROADS simulation model online and used it during task 2. This group probably had the best experience. Thus, it would be helpful to encourage participants to use the online simulation model and, more generally, to encourage active simulation.

Overall, there is some room for improvement, but for the first online workshop, it worked well. Students seemed to appreciate the online session despite it being unfamiliar to them.

### Group model building and prioritisation workshops on the long‐term quality of the blue, green, and built infrastructure

A series of three GMB workshops plus a prioritisation workshop were held as part of a larger participatory collaboration with stakeholders about the Thamesmead region south‐east of London. The Thamesmead region is characterised by high levels of social housing, a large Black community, future improved connectivity to the public transport network, and plans to build 20,000 new homes. Between April and June 2020, two members of the London‐based team conducted a series of three short GMB workshops online, which replaced a full‐day in‐person workshop with one of our stakeholder subgroups. The series of GMB workshops focused on the shared concern: “to sustain and increase the quality of the Built, Blue and Green environment to ensure long term stewardship in Thamesmead.” They aimed to map the stakeholder subgroup's system boundary and understanding of how quality and stewardship can be enhanced. The online series of workshops involved a small group of five stakeholders from the same organisation in total, with two to four attending each individual workshop. Stakeholders and facilitators attended online via individual computers. After an introduction of the purpose of the meeting, a brief introduction about CLDs at the first workshop or a recap at subsequent workshops, the main part of the workshops was devoted to GMB activities (see *Table* 4). One facilitator acted as the modeller and shared her screen with Vensim modelling software open and a second acted as the main facilitator, with the modeller intervening as questions arose. Details are described in the online supplement.

**Table 4:** Participant agenda of the Thamesmead group model‐building workshops

Thamesmead group model‐building workshops (2 hours each session)	
10 minutes	Introduction (welcome and workshop objectives, recap of previous work/session)
20 minutes	(Short) variable elicitation session (in 1st session)
85 minutes	Creating a causal loop diagram (105 minutes in 2nd and 3rd session)
5 minutes	Closing and next steps

In July 2020, the same facilitator and modeller and an additional new member from the London team conducted a prioritisation workshop online with a more diverse group of 12 participants representing seven organisations. This workshop served to present and discuss subgroups' similarities and differences concerning the problem boundaries and agree on a prioritised starting point for quantitative modelling. The workshop agenda is shown in *Table* 5.

**Table 5:** Participant agenda of the Thamesmead prioritisation workshop

Thamesmead prioritisation workshop (3 hours)	
5 minutes	Relevant sectors poll – part 1 while participants arrive
20 minutes	Introduction
30 minutes	System boundaries ‐ Presentation of the results
25 minutes	System boundaries – Free discussion session
5 minutes	*Break*
30 minutes	System boundaries – Focused discussion session and wrap‐up
15 minutes	Relevant sectors poll – part 2
40 minutes	Focus of the simulation – Discussion
5 minutes	Closing and next step
5 minutes	Evaluation

We mostly used Microsoft Teams software for these workshops but switched to Zoom software one time because of stakeholder preferences. In addition, the facilitators used a WhatsApp software channel to communicate privately with each other at the prioritisation workshop.

The lessons learned from running the workshops online were as follows:

- *Workshop format*: During the GMB workshops, having everybody sit by themselves in front of their computer screen allowed stakeholders to be very focused on the model structure. Participants had time to reflect on variables and links. We thus preferred this over a hybrid setting. At the prioritisation workshop, we preferred to communicate project results simply and quickly in order to provide more time for the group discussion.
- *Number of participants*: The GMB workshops had the ideal number of participants (two to four) for an online GMB activity. It allowed them to be free to talk when they wanted to say something. As it was a small‐group setting, it did not feel like we were lacking the social component. Participants worked in the same organisation and had already gotten used to online meetings during the lockdown. However, at the prioritisation workshop with 12 participants, a subgroup showed occasional signs of imbalanced contribution or distraction. The workshops allowed us to add several silent observers for note taking and learning purposes. Running GMB and prioritisation workshops with a large group would be more difficult because it would require more formal rules for participation (e.g. by raising hands) as well as more monitoring by a process facilitator (Richardson and Andersen, 1995) to ensure relatively equal contribution.
- *Workshop length*: The three GMB workshops had the ideal duration (2 hours). The prioritisation workshop had a good length (3 hours) and included a break, which helped everybody keep focused.
- *Participants' focus*: The online setting seemed to enhance participant focus for the very small groups, but not as strongly in the midsize group, where there was more opportunity for distraction. Similar to in‐person settings, the importance of process facilitation to ensure equal participation and focus seemed to grow with group size. The use of webcams created a personal atmosphere and also seems a good recommendation for giving a sense of actual interest and participation.
- *Dedicated communication platform for facilitators*: While the small GMB workshops did not require any separate communication between the facilitators, the larger number of 12 participants at the prioritisation workshop and greater uncertainties with regards to the prioritised content made a separate communication channel crucial. It allowed the facilitation process to be adjusted to the context and to better manage the group discussion. We thus recommend this for larger groups, difficult settings, or facilitator teams who are not yet very experienced in collaborating with each other.

Overall, the team felt that the setting worked very well and participants emphasised their appreciation of the online activities. We could even increase participation by holding workshops online. This is likely because of the shorter duration and because the online setting did not require participants to travel, which easily takes 30–90 minutes one way in London.

## Discussion and conclusions

This article presented three rather diverse cases of moving system dynamics workshops online. They include a broad spectrum of participatory online workshop activities and purposes ranging from using an existing simulation model to improving CLDs, to building CLDs from scratch. The article showed that conducting these diverse workshop activities virtually is feasible and can often work similarly well as in‐person workshops. The examples covered diverse research and teaching settings. They also included different types of online participation. The En‐ROADS Climate Workshop, the GMB workshops, and the prioritisation workshops were exclusively online with everybody participating via their own computer or tablet. The tower refurbishment workshops were of hybrid nature: the first workshop brought the facilitator in online, and the second workshop incorporated some online participants into an otherwise in‐person setting. This shows that the system dynamics activities are both highly adaptable and resistant to the varying nature of specific circumstances. We found the fully online workshops easier to run, and future research could thus put special emphasis on exploring how to fruitfully collaborate in hybrid settings.

Our cases used diverse online meeting platforms, and we found that virtual system dynamics workshops can work well using meeting software with basic audio‐visual and chat functions together with basic system dynamics software functionality on the modeller's computer, given stable Internet connections. A hands‐up feature can become useful in larger group settings. Whiteboard features may enhance the possibility to effectively collaborate, with all participants actively pointing to and/or modifying what is shared. However, it is important to consider user friendliness and participants' online literacy and access to and familiarity with a certain platform. We also found the video‐recording feature of the platforms useful because it allows us to easily go back into the workshop setting for reporting and research purposes.

We found that small and short workshops led to more fruitful engagement. Thus, dividing a full‐day workshop into a sequence of several individual workshops is effective. In the larger and more complex settings of the prioritisation workshop, we found an external communication channel among the facilitators very useful. It cannot replace the exchange of diverse cues between the facilitators, e.g. eye contact, confirmatory nodding and smiling, or a blank look and request for help. But together with strategically placed breaks, it is an improvement over no communication.

Some of the workshops brought together groups from different countries. Online workshops may thus not only be a temporary solution during COVID‐19 times, but they can serve the purpose of bringing together geographically dispersed participants more generally (see also Yearworth and White, 2019). Even for stakeholders located in a similar geographical region, the ability to participate online seems to have increased participation. This can make online workshops a valid option in certain settings because of their positive implications on travel needs and time effort, emissions, and climate change.

Our cases thus show that system dynamics scripts allow for resilient workshop settings, including online workshops. Thus, we recommend conducting system dynamics workshops virtually, when needed. This could help establish a similar knowledge base as we have for in‐person settings. To build up such a knowledge base, it would be useful to further address questions related to the design and impact of virtual workshops and engagements. Specific questions for further research that our three cases sparked for the system dynamics and wider research community are listed below.

Workshop design:

- How can large GMB workshops be facilitated online? In particular, how can workshops be designed so that everybody can contribute to model building, feels engaged and listened to, and so that a process coach (Richardson and Andersen, 1995) or facilitator can observe social cues even in large groups?
- Which workshop designs are recommended in a hybrid setting with some participants or facilitators sharing physical space?
- How can GMB and other facilitated systems dynamics workshops be organised when it is difficult to divide a long workshop into different sessions?
- How can participant contribution be diversified, e.g. moving from mostly verbal contributions to simultaneous collaboration on a whiteboard, etc.? In particular, how does one familiarise participants quickly with used software, enhance sustained attention to the screen, balance presentations (to share results) and interactive sessions to sustain attention, or move to synchronous interaction of participants, e.g. in addition to the time‐demanding one‐by‐one inputs used by us and the simultaneous process used by Wilkerson *et al*. (2020)?

Workshop impact:

- Do virtual workshops differ in their effects on communication, insights, and decision‐making from in‐person settings – and if so, how and why?
- How green are virtual workshops, e.g. when the emissions from data sharing are offset against those from reduced travel needs?

## Supporting information


**Appendix S1.** Supporting Information

## Footnotes

i
Author involvement in workshops: first Tower Refurbishment workshop: NZ, MT, HS, IH, TK; second Tower Refurbishment workshop: NZ, KD, TC, MT, HS, IH, TK; teaching case: NZ; Thamesmead group model‐building workshops: IP, NZ; Thamesmead prioritisation workshop: IP, GS, NZ.
