# Supplementary material for: Moving online: reflections from conducting system dynamics workshops in virtual settings
Source: Syst Dyn Rev. 2020 Dec 1;37(1):59–71. doi: 10.1002/sdr.1667 (PMC8246820; doi:10.1002/sdr.1667)
Supplement: Supplementary file 1 — Appendix S1. Supporting Information [file SDR-37-59-s001.pdf]

ONLINE SUPPLEMENT

TABLE OF CONTENTS

Confirmatory/Disconfirmatory Model Validation with Online Participation: Best Practices Guidelines for Tower Block Refurbishment . 2
Participant agenda 2
Workshop facilitation manual 3
Workshop aim 3
Time 3
Preparation for the workshop 3
Inputs 3
Outputs 3
Roles 4
Instructions for the ‘small group work on CLDs’ sub-sessions for the project partners who participate online as expert participants: 4
Detailed agenda 4
Group Model Building: Thamesmead 6
Thamesmead system dynamics workshop (TM SD WS) – Causal loop diagram (CLD) – 1st part 6
Workshop aim 6
Materials 6
To do list 6
Detailed agenda 7
Thamesmead system dynamics workshop (TM SD WS) – Causal loop diagram (CLD) – 2nd part 10
Detailed agenda 10
Thamesmead system dynamics workshop (TM SD WS) – Causal loop diagram (CLD) – 3rd part 12
Detailed agenda 12
References 14

CONFIRMATORY/DISCONFIRMATORY MODEL VALIDATION WITH ONLINE PARTICIPATION: BEST PRACTICES GUIDELINES FOR TOWER BLOCK REFURBISHMENT

Date: 27 June 2019
Time: 13:00–16:00
Location: UCL plus online participation

Participant agenda

Start	End	Activity
13:00	13:10	Introduction of participants
13:10	13:20	Presentation of the project and intermediate results
13:20	14:00	Small group work on causal diagrams<br>10 min presentation of a diagram<br>30 min improvement of the diagram
14:00	14:30	Presentation of the results to the plenary and discussion
14:30	14:40	Break
14:40	15:20	Small group work on causal diagrams<br>10 min presentation of a diagram<br>30 min improvement of the diagram
15:20	15:50	Presentation of the results to the plenary and discussion
15:50	16:00	Discussion and close

Workshop facilitation manual

Workshop aim

This workshop setting serves validation purposes and presents an existing causal loop diagram (CLD) to participants and asks them for structure confirmation or improvement and/or adaptation to a slightly different (e.g. geographical) context. As a special feature, it includes a mix of in-person and online participation. It is informed by the disconfirmatory interviews (Andersen et al., 2012) and the Model Review script (Scriptapedia Wikibooks contributors, no year).

Time

3 hours for the entire workshop

Preparation for the workshop

Presentations	Participation	Setting	Materials
• Introductory slides<br>• Unfolding CLD presentations	• Consent forms<br>• Information sheets	• Group tables<br>• Videoconferencing set-up	• Posters with CLDs<br>• Sturdy tape<br>• Audio recorders on tables<br>• A4 paper on tables<br>• Pens on tables<br>• Flipchart markers on table<br>• Whiteboard markers in room

Inputs

CLDs

Outputs

Improved CLDs, shared understanding of changes

Roles

Main facilitator, facilitator for each sub-group (including main facilitator), online expert participants

Instructions for the 'small group work on CLDs' sub-sessions for the project partners who participate online as expert participants:

- If participants have questions, you can act as an expert because the facilitators may not know all the background information
- Please act as a non-dominant participant. This means, you can use your expertise to sometimes come into the conversation, or to make us aware if you think we focus on minor details and not the important aspects, etc.

Detailed agenda

Start	End	Activity	Led by	Explanation
13:00	13:10	Introduction of participants	Main facilitator	
13:10	13:20	Presentation of the project and results	Main facilitator	
13:20	14:00	Small group work on CLDs<br>10 min presentation of a diagram<br>30 min improvement of the diagram	All facilitators	each group works on one of the CLDs<br><br>Sub-group facilitator presents the CLD to the group (3 min) and answers questions (7 min)<br><br>Facilitator asks whether participants agree with the CLD, whether it mis-represents certain areas, whether important areas are missing<br><br>Facilitator asks whether other group members agree or disagree with the suggestions, facilitator may also suggest re-phrasing of variable names<br><br>Facilitator then invites the participant to make adjustments to the CLD<br><br>CLD amendments continue<br><br>Facilitators need to make sure not to get hold up with just one issue for two long
14:00	14:30	Presentation of the results to the plenary and discussion	All facilitators + group member	For each group, one group member and facilitator present the results (Facilitators let the group member start and chip in to clarify details if necessary)<br>- Main idea of the diagram<br>- Main improvements
14:30	14:40	Break		

14:40	15:20	Small group work on CLDs<br>10 min presentation of a diagram<br>30 min improvement of the diagram	All facilitators	same groups, each group works on a different CLD now<br><br>Sub-group facilitator presents the CLD to the group (3 min) and answers questions (7 min)<br><br>Facilitator asks whether participants agree with the CLD, whether it mis-represents certain areas, whether important areas are missing<br><br>Facilitator asks whether other group members agree or disagree with the suggestions, facilitator may also suggest re-phrasing of variable names<br><br>Facilitator then invites the participant to make adjustments to the CLD<br><br>CLD amendments continue<br><br>Facilitators need to make sure not to get hold up with just one issue for two long
15:20	15:50	Presentation of the results to the plenary and discussion	All facilitators + group member	For each group, one group member and facilitator present the results (Facilitators let the group member start and chip in to clarify details if necessary)<br>- Main idea of the diagram<br>- Main improvements
15:50	16:00	Discussion and close	Main facilitator	

GROUP MODEL BUILDING: THAMESMEAD

Thamesmead system dynamics workshop (TM SD WS) – Causal loop diagram (CLD) – 1st part

Date: 7 April 2020
Time: 14:00–16:00
Location: online

Workshop aim

The WS will focus on building of a Thamesmead causal map around the identified shared concern (identified at previous problem scoping WS): **“To sustain and increase the quality of Built (i) and Blue/Green (ii) environment to ensure long term stewardship”.** The specific sub-themes identified were: (i) Connectivity and (multi) use of canals for the blue and green spaces (ii) Housing (this issue has been identified but not discussed in detail).

Materials

Two audio recorders (batteries)	As backup to online recording

To do list

Status	Actions	Comments
✓	Call/email participants for confirmation	
✓	Send consent forms and information sheets	
✓	Send evaluation form	
✓	Teams link	
✓	Guidance for observers	
✓	Find out how to save in the online meeting platform and what the best way is to make the variable list available during modelling	
✓	Find out how we can record the session and have a video available to save on a computer	
✓	Download the recording	

Detailed agenda

Time	Activity	Led by	Description	Objective	Outcome	Preparation
pre-WS		Modeller	• Sign the consent form via email<br>• Email asking 3 variables defining long term quality of the Built, Blue and Green environment	To help the variable elicitation		
14:00 - 14:10	Introduction	Facilitator	• Welcome and overview of the workshop agenda and objectives<br>• **FACILITATOR ASKS FOR CONSENT**<br>• **FACILITATOR STARTS RECORDING**<br>• Brief introductions from participants: presentation of their work on Thamesmead<br>• **Short presentation of the researchers actively involved today: facilitator + modeller + 2 note takers. "Due to the lack of time, the researchers online will take notes and otherwise be silent observers. We hope to have a more relaxed workshop in the near future.**"<br>• Brief recap of the problem identification workshop, the shared concern, the clusters and the problems map (i.e. the work that preceded this workshop)	Warm up for orientation, trust-building and goal clarification<br>Background on SD.		- Slides
14:10 - 14.30	Variable elicitation (short)	Facilitator<br>Modeller	• The facilitator explains the task and the task-focusing question: "We will try to understand how you define the quality of the Built and Blue/Green environment to ensure long term stewardship. What key variables do we need to consider in order to understand the quality of the Blue, Green and Built environment?"<br>• The facilitator explains that the goal of this activity is to come up with the variables that play a key role in this issue and to write them into the shared Vensim screen. Participants can improve each other's variable names.<br>• The modeler shares the screen with Vensim open.<br>• Participants are asked to report the variables defining their understanding of "quality" (also its causes).	Core variable elicitation	Definition of quality according to different group of stakeholders (this will possibly be used as decision criteria or co-benefits)	- Slide activity<br>- Computer running modelling software (Vensim)<br>- Screen shared

Time	Activity	Led by	Description	Objective	Outcome	Preparation
			• When a variable name is open to several interpretations, the facilitator asks for a brief description or definition of the variable, including the units in which the variable can be measured.<br>• **No discussion to save time, a dry list.**<br>• Once all variables have been shared, the wall builder reflects back the themes that emerged from wall-building in Vensim.			
14:30<br>-<br>15:50	Creating Causal Loop Diagram<br>–<br>Session 1	facilitator, modeller	• Starting from the variables defining quality of the built/blue/green space and long term stewardship, we will create a causal map, underlining mechanism that represents driver/barrier to the long term quality. The aim is to understand the causal interconnection between the elements mentioned before.<br>• The modeler shares the screen.<br>• The facilitator introduces the exercise by reviewing the variable list.<br>• *"The CLD will be built identifying hypothesized causal relationships between variables. These connections can be based on the literature, your own research or conjectures."*<br>• Begin by picking variables that are important and arranging them on the whiteboard and then drawing a casual arrow from the cause to the effect. Then add a '+' or '-' sign to indicate the direction of influence with '+' signs representing change in the same direction, and '-' signs representing change in the opposite direction.<br>(A '+' sign indicates that increasing one leads to an increase in the other, and a decrease in one leads to a decrease in the other. A '-' sign indicates an opposite effect where increasing one leads to a decrease in the other, and a decrease in one leads to an increase in the other.)<br>• As someone suggests something, the modeler draws the link on the model. The facilitator will then encourage participants	To build the Thamesmead CLD	Thamesmead conceptual model	- Slide activity<br>- Computer running modelling software (Vensim)<br>- Screen shared<br>- Core variables visible on the side of the Vensim page

Time	Activity	Led by	Description	Objective	Outcome	Preparation
			to add variables and relationships. The modeler tries to get things recorded using participants' terms.<br>• (if needed) The facilitator asks questions that help identify impact and causal relations between identified key variables. As the number of links increases, look for feedback loops.<br>• If the group begins to slow down and there is time, or no feedback loop has been formed, the facilitator will ask if there are any relationships between the identified variables that have not been discussed. Doing this will help create loops that might otherwise have been missed.			
15:50 - 16:00	Closing and next step	Facilitator	• Summary<br>• Next steps		Next steps	- Slide next steps
Post-WS		Modeller	• Evaluation form via email			- Evaluation form

Thamesmead system dynamics workshop (TM SD WS) – Causal loop diagram (CLD) – 2nd part

Date: 22 April 2020
Time: 12:00–13:30
Location: online

Detailed agenda

Time	Activity	Led by	Description	Objective	Outcome	Preparation
12:00<br>-<br>12:10	Introduction	Facilitator	• Welcome and overview of the workshop agenda<br>• **FACILITATOR ASKS FOR THE CONSENT**<br>• **FACILITATOR STARTS RECORDING**<br>• IF NEEDED - Brief introductions from new participants: presentation of their work on Thamesmead.<br>• Recap of the previous modelling session<br>• **IF NEEDED - Short presentation of the CAMELLIA/CUSSH researchers actively involved today: facilitator + modeller + note takers. "Due to the lack of time, the researchers online will be silent observers. We hope to have a more relaxed workshop in the near future."**)	Warm up for orientation, trust-building and goal clarification<br>Recap from CLD building session 1.		- model on the screen
12:10<br>-<br>13:25	Creating Causal Loop Diagram<br>-<br>Session 2	Facilitator<br>Modeller	• Starting from the variables defining quality of the built/blue/green space and long term stewardship, we will create a causal map, underlining mechanism that represents driver/barrier to the long term quality. The aim is to understand the causal interconnection between the elements mentioned before.<br>• The modeler shares the screen<br>• The facilitator introduces the exercise by reviewing the variable list.<br>• *"The CLD will be built identifying hypothesized causal relationships between variables. These connections can be based on the literature, your own research or conjectures."*	To build the Thamesmead CLD, Group 3	Thamesmead conceptual model	- Computer running modelling software (Vensim)<br>- Screen shared<br>- Core variables visible on the side of the Vensim page

Time	Activity	Led by	Description	Objective	Outcome	Preparation
			• Begin by picking variables that are important and transferring them to the whiteboard and then drawing a casual arrow from the cause to the effect. Then add a '+' or '-' sign to indicate the direction of influence with '+' signs representing change in the same direction, and '-' signs representing change in the opposite direction.<br>(A '+' sign indicates that increasing one leads to an increase in the other, and a decrease in one leads to a decrease in the other. A '-' sign indicates an opposite effect where increasing one leads to a decrease in the other, and a decrease in one leads to an increase in the other.)<br>• As someone suggests something, the modeler draws the link on the model. The facilitator will then encourage participants to add variables and relationships. The modeler tries to get things recorded using exactly the same terms as the participants.<br>• (if needed) The facilitator asks questions that help identify impact and causal relations between identified key variables. As the number of links increases, look for feedback loops.<br>• If the group begins to slow down and there is time, or no feedback loop has been formed, the facilitator will ask if there are any relationships between the identified variables that have not been discussed. Doing this will help create loops that might otherwise have been missed.			
13:25 - 13:30	Closing	Facilitator Modeller	• Closing<br>• Modeller evaluation form – link in the chat		Next steps	- Evaluation form
Post-WS		Modeller	• Evaluation form via email			- Evaluation form

Thamesmead system dynamics workshop (TM SD WS) – Causal loop diagram (CLD) – 3rd part

Date: 5 June 2020
Time: 10:00–12:00
Location: online

Detailed agenda

Time	Activity	Led by	Description	Objective	Outcome	Preparation
10:00<br>-<br>10:15	Introduction	Facilitator	• Welcome and overview of the workshop agenda<br>• **FACILITATOR ASKS FOR THE CONSENT**<br>• **FACILITATOR STARTS RECORDING**<br>• Recap of the previous modelling sessions<br>• IF NEEDED - Brief introductions from new participants: presentation of their work on Thamesmead.<br>• **IF NEEDED - Short presentation of the CAMELLIA/CUSSH researchers actively involved today: facilitator + modeller + note takers. "Due to the lack of time, the researchers online will be note takers and otherwise silent observers."**)	Warm up for orientation, trust-building and goal clarification<br>Recap from CLD building session 1.		- model on the screen<br>- prepare the questions for the recap
10:15<br>-<br>11:55	Creating Causal Loop Diagram<br>-<br>Session 3	Facilitator<br>Modeller	• Starting from the variables defining quality of the built/blue/green space and long term stewardship, the aim is to create a causal map, underlining mechanisms that represent drivers/barriers to the long term quality. The aim is to understand the causal interconnection between the elements mentioned before.<br>• The modeler shares the screen<br>• The facilitator introduces the exercise by reviewing the variable list.<br>• *"The CLD will be built identifying hypothesized causal relationships between variables. These connections can be based on the literature, your own research or conjectures."*<br>• Begin by picking variables that are important and transferring them to the whiteboard and then drawing a	To build the Thamesmead CLD, Group 3	Thamesmead conceptual model	- Computer running modelling software (Vensim)<br>- Screen shared<br>- Core variables visible on the side of the vensim's page

Time	Activity	Led by	Description	Objective	Outcome	Preparation
			casual arrow from the cause to the effect. Then add a '+' or '-' sign to indicate the direction of influence with '+' signs representing change in the same direction, and '-' signs representing change in the opposite direction.<br>(A '+' sign indicates that increasing one leads to an increase in the other, and a decrease in one leads to a decrease in the other. A '-' sign indicates an opposite effect where increasing one leads to a decrease in the other, and a decrease in one leads to an increase in the other)<br>• As someone suggests something, the modeler draws the link on the model. The facilitator will then encourage participants to add variables and relationships. The modeler tries to get things recorded using exactly the same terms as the participants.<br>• (if needed) The facilitator asks questions that help identify impact and causal relations between identified key variables. As the number of links increases, look for feedback loops.<br>• If the group begins to slow down and there is time, or no feedback loop has been formed, the facilitator will ask if there are any relationships between the identified variables that have not been discussed. Doing this will help create loops that might otherwise have been missed.			
11:55 - 12:00	Next step and Closing	Facilitator Modeller	• Next steps:<br>• **residents, introduce us to the residents (local community leader) for interview and suggest social media groups**<br>• **Local authorities: should we try to invite them for the July workshop? If yes, we ask for their contact/name directly.**<br>• **July workshop – link doodle to find date**<br>• Closing<br>• Modeller evaluation form – link to be copied in the chat		Next steps	- Evaluation form
Post-WS		Modeller	• Evaluation form via email (opportunity if not filled during workshop)			- Evaluation form

References

Andersen, D.L., Luna-Reyes, L.F., Diker, V.G., Black, L., Rich, E., Andersen, D.F., 2012. The disconfirmatory interview as a strategy for the assessment of system dynamics models. System Dynamics Review 28, 255–275.

Scriptapedia Wikibooks contributors, no year. Scriptapedia. Wikibooks, The Free Textbook Project, https://en.wikibooks.org/wiki/Scriptapedia.
